# A randomised assessment of image guided radiotherapy within a phase 3 trial of conventional or hypofractionated high dose intensity modulated radiotherapy for prostate cancer

**DOI:** 10.1016/j.radonc.2019.10.017

**Published:** 2020-01

**Authors:** Julia Murray, Clare Griffin, Sarah Gulliford, Isabel Syndikus, John Staffurth, Miguel Panades, Christopher Scrase, Chris Parker, Vincent Khoo, Jamie Dean, Helen Mayles, Philip Mayles, Simon Thomas, Olivia Naismith, Angela Baker, Helen Mossop, Clare Cruickshank, Emma Hall, David Dearnaley, on behalf of CHHiP Investigators

**Affiliations:** a The Institute of Cancer Research, London, UK; b Royal Marsden NHS Foundation Trust, London, UK; c Department of Radiotherapy, University College London Hospitals NHS Foundation Trust, UK; d Clatterbridge Cancer Centre, Wirral, UK; e Cardiff University/Velindre Cancer Centre, UK; f Lincoln County Hospital, UK; g Ipswich Hospital, UK; h Addenbrooke’s Hospital, Cambridge, UK; i Royal Berkshire Hospital, Reading, UK

**Keywords:** Prostate, Image-guided radiotherapy, Toxicity, Dosimetry

## Abstract

**Background and purpose:**

Image-guided radiotherapy (IGRT) improves treatment set-up accuracy and provides the opportunity to reduce target volume margins. We introduced IGRT methods using standard (IGRT-S) or reduced (IGRT-R) margins in a randomised phase 2 substudy within CHHiP trial. We present a pre-planned analysis of the impact of IGRT on dosimetry and acute/late pelvic side effects using gastrointestinal and genitourinary clinician and patient-reported outcomes (PRO) and evaluate efficacy.

**Materials and methods:**

CHHiP is a randomised phase 3, non-inferiority trial for men with localised prostate cancer. 3216 patients were randomly assigned to conventional (74 Gy in 2 Gy/fraction (f) daily) or moderate hypofractionation (60 or 57 Gy in 3 Gy/f daily) between October 2002 and June 2011. The IGRT substudy included a second randomisation assigning to no-IGRT, IGRT-S (standard CTV-PTV margins), or IGRT-R (reduced CTV-PTV margins). Primary substudy endpoint was late RTOG bowel and urinary toxicity at 2 years post-radiotherapy.

**Results:**

Between June 2010 to July 2011, 293 men were recruited from 16 centres. Median follow-up is 56.9(IQR 54.3–60.9) months. Rectal and bladder dose-volume and surface percentages were significantly lower in IGRT-R compared to IGRT-S group; (p < 0.0001). Cumulative proportion with RTOG grade ≥ 2 toxicity reported to 2 years for bowel was 8.3(95% CI 3.2–20.7)%, 8.3(4.7–14.6)% and 5.8(2.6–12.4)% and for urinary 8.4(3.2–20.8)%, 4.6(2.1–9.9)% and 3.9(1.5–9.9)% in no IGRT, IGRT-S and IGRT-R groups respectively. In an exploratory analysis, treatment efficacy appeared similar in all three groups.

**Conclusion:**

Introduction of IGRT was feasible in a national randomised trial and IGRT-R produced dosimetric benefits. Overall side effect profiles were acceptable in all groups but lowest with IGRT and reduced margins.

**ISRCTN:**

97182923.

## Highlights

• Introduction of daily online prostate IGRT was feasible in a national randomised trial.
• Dosimetric benefits seen in patients treated with IGRT and reduced CTV-PTV margins.
• Overall side effect profiles were acceptable in all groups but lowest with IGRT and reduced margins.

Intensity-modulated radiotherapy (IMRT) enables dose escalation to the prostate target volume, with low gastrointestinal or genitourinary toxicity [1], [2], [3], [4]. The success of radical prostate radiotherapy depends on accurate delivery of high dose conformal radiotherapy to a defined target volume. Image guided radiotherapy (IGRT) with daily online imaging has the potential to improve prostate localisation, consequently improving treatment accuracy and reducing the required clinical (CTV) to planning (PTV) target volume margin [5]. This may reduce the amount of normal tissue receiving target doses, and consequently toxicity [6]. Intrafraction motion and outlining uncertainties still necessitate a small margin around the CTV [5].

In addition to optimising prostate radiotherapy techniques, there has been interest in the exploitation of fraction sensitivity of prostate cancer through hypofractionation [7], [8], [9]. This has been successfully examined within the UK multicentre randomised controlled trial (Conventional or Hypofractionated High-dose Intensity Modulated Radiotherapy in Prostate Cancer; CHHiP) which aimed to compare the efficacy and toxicity of conventional and hypofractionated radiotherapy using high-quality radiation techniques. Within the trial, 3216 patients were enrolled from 71 centres within the UK between October 2002 and June 2011 [10].

During the latter stages of the CHHiP trial, IGRT became available in participating treatment centres. To assess this technology the CHHiP IGRT phase 2 substudy was developed. We aimed to determine the feasibility and generalisability of IGRT in the context of a multicentre trial and assess acute and late toxicity. A patient reported outcome (PRO) protocol was subsequently integrated into the substudy. To our knowledge, this is the only randomised study undertaken evaluating daily prostate image-guided IMRT with and without reduced PTV margins.

## Materials and methods

### Study design and participants

CHHiP is a randomised phase 3, non-inferiority trial which recruited men with localised prostate cancer (pT1b-T3aN0M0) [10]. Patients were randomly assigned (1:1:1) to conventional 74 Gray (Gy) in 2 Gy/fraction (f) daily or one of two hypofractionated schedules giving 60 Gy or 57 Gy in 3 Gy/f daily. Patients were all treated with IMRT [11].

The IGRT substudy was approved by Central London REC1 Research Ethics Committee (10/H0718/31) and implemented in June 2010. Men who had entered the CHHiP trial were eligible for the IGRT substudy with additional consent and provided they had no contraindication to implanted fiducial markers or a hip prosthesis or fixation which would interfere with positional imaging. Following dose/fractionation randomisation in the main CHHiP trial, minimisation was used to assign IGRT substudy patients in a 1:1:1 ratio to either no-IGRT – using standard CHHiP planning margins, IGRT using standard CHHiP planning margins (IGRT-S), or IGRT with reduced planning margins (IGRT-R). Radiotherapy centre and dose/fractionation schedule were used as balancing factors. Neither patients nor clinicians were blinded to allocation. Sixteen UK radiotherapy centres took part in the IGRT substudy. Centres could choose, depending on previous IGRT experience, to randomise to all three treatment technique options or to the 2-way randomisations: no IGRT versus IGRT-S or IGRT-S versus IGRT-R. Four centres used the 3-way randomisation, five centres randomised to no-IGRT vs IGRT-S and seven centres randomised to IGRT-S vs IGRT-R. In 2014, a patient reported outcomes (PRO) assessment was introduced to collect data at a single time point at least three years post randomisation. This separate protocol received ethical approval from the NRES Committee South West – Central Bristol (14/SW/1071).

### Treatment

Patients randomised to treatment with IGRT, either had fiducial markers inserted into the prostate using trans-rectal ultrasound guidance or soft tissue matching if using the TomoTherapy**®** system. Fiducial markers were implanted with antibiotic cover approximately 2 weeks prior to the radiotherapy planning scan. Patient positioning was supine and target and treatment planning volumes have been previously described [11]. Radiotherapy was planned and delivered using an integrated simultaneous boost technique (SIB) with three different target volumes and dose levels as previously detailed [12] and illustrated in Table S1. In the no-IGRT and IGRT-S arms the standard CHHiP CTV to PTV posterior margins of 10 mm/5 mm/0 mm were used. In the IGRT-R arm, these posterior margins were 6 mm/3 mm/0 mm. Mandatory dose constraints were defined for both target coverage and avoidance of normal tissues including rectum, bowel, bladder and femoral heads (Table S2). Treatment was delivered with 6–15 MV photons with multileaf collimators to shape beams.

Patients randomised to no-IGRT had offline portal imaging to verify treatment accuracy; the match to bony landmarks was to be within 3 mm. Patients receiving IGRT had daily pre-treatment imaging and any observed set-up error ≥2 mm was corrected prior to treatment. No post-correction imaging was taken. A prospective quality-assurance programme was designed as an integral part of the study [13] including specific aspects for the IGRT substudy (Supplementary material).

### Trial assessments

Pre-trial staging investigations included PSA, lymph node assessment by MRI or CT, and bone scan. Histology was assessed from diagnostic TRUS guided biopsies (or TURP specimens) and reported using the Gleason system.

Toxicity experienced from fiducial marker insertion was recorded using CTCAE grading [14].

Pre-hormone and pre-radiotherapy clinical assessments used Late Effects of Normal Tissues Subjective-Objective Management (LENT-SOM) [15] and the Royal Marsden Hospital (RMH) grading [16]. Clinical assessment of acute toxicity was made weekly during radiotherapy and at weeks 10, 12 and 18 from the start of radiotherapy using the Radiation Therapy Oncology Group (RTOG) scoring system [17]. Late toxicity was assessed at 6, 12, 18 and 24 months then annually to 5 years using RTOG, LENTSOM and RMH scoring systems.

In the PRO substudy, data was collected at a single time point using the Expanded Prostate Cancer Index Composite (EPIC) questionnaire, (EPIC-50 used for bowel and urinary domains and EPIC-26 for sexual and hormonal domains) [18], the Vaizey Incontinence [19], Short Form 12 (SF-12) [20] and International Index of Erectile Function (IIEF-5) [21] questionnaires. Questionnaires were sent directly from participating centres to patients (following confirmation of health status) who were at least three years from completing treatment. A single reminder letter was sent.

For each patient, treatment planning data (planning CT, dose distribution and organ contours) were uploaded using dedicated analysis software (VODCA. MSS Medical Software Solutions, Hagendorn, Switzerland). Using in-house code, all radiotherapy plans were converted into equivalent dose in 2 Gy per fraction, using Withers formula [22] with an α/β ratio of 3 Gy for rectum and 5 Gy for bladder for each dose cube voxel. Dose volume (DVH) and dose-surface (DSH) histograms were generated for rectum and bladder.

### Statistical considerations

The IGRT substudy was non-comparative and powered to assess toxicity independently within each treatment technique group using Simon single stage design with exact p-values [23]. The primary endpoint was proportion of patients with RTOG bladder or bowel toxicity of grade ≥2 at two years from starting radiotherapy. Secondary endpoints included acute toxicity, prevalence of late radiation induced toxicity, time to late radiation induced toxicity, toxicity associated with fiducials and feasibility of delivery of IGRT in a multi-centre setting. Efficacy has been included as exploratory analyses. Ninety-one patients were required (with 79 or more remaining toxicity-free) in each group to give 80% power to detect a 10% RTOG bladder/bowel grade ≥2 toxicity rate at 2 years with IGRT assuming a 20% toxicity rate with no IGRT (alpha 3.4%). Sample size was not calculated for the PRO substudy, all eligible IGRT substudy patients were invited to participate.

### Analysis methods

All analyses have been presented according to randomly allocated treatment technique group. Analyses of side effects included all data available at each time point for patients who received at least one fraction of radiotherapy (unless otherwise stated). Worst acute bladder and bowel toxicity was calculated using worst grade reported during the first 18 weeks from start of radiotherapy. For the primary endpoint, only patients with a 2-year RTOG toxicity assessment were included in the denominator, although a sensitivity analysis was conducted using all randomised patients. The proportion of patients with RTOG bladder or bowel toxicity of grade ≥2 at 2 years were presented together with exact binomial 95% confidence intervals. Time to first occurrence of late radiation induced side effects were analysed using Kaplan Meier method to calculate the cumulative proportion with events reported on 2-year assessment form for each scoring system. Time was measured from start of radiotherapy. Patients not experiencing an event were censored at date of last toxicity assessment or at date of death for deceased patients. The log-rank test was used to compare no IGRT versus IGRT-S and IGRT-S versus IGRT-R with a significance level of 1%, to account for multiple comparisons. Biochemical/clinical failure was defined as time to first PSA failure (PSA value greater than nadir +2 ng/ml with a consecutive confirmatory PSA value) or prostate cancer recurrence (local, lymph node, pelvic or distant). Patients event free at the time of analysis were censored at their last know PSA assessment.

Statistical analyses were based on a data snapshot taken on 18th May 2016 (except for efficacy analyses which were based on a snapshot taken on 3rd April 2018 to maximise data maturity). All analyses were performed using STATA Version 13.1. Patient reported outcomes were scored in accordance with the recommended scoring manuals [24], [25] and presented as descriptive statistics by treatment group. The Vaizey questionnaire is scored on a continuous scale, with minimum score, 0 representing perfect continence and a maximum score, 24 representing total incontinence [19]. Patients were divided into 3 categories for Vaizey total score according to tertiles and dose data presented. In health related quality of life, the clinically meaningful change is defined as a mean change score exceeding half the standard deviation of baseline value [26]. As there was no baseline data available for this patient group, the mean and standard deviation values from the main CHHiP trial QoL substudy [27] (Table S3) were used to define a threshold score for a meaningful change for the EPIC bowel and urinary domain scores.

## Results

Two-hundred and ninety-three patients (48 no-IGRT, 137 IGRT-S and 108 IGRT-R) from 16 radiotherapy centres across the UK were randomised between July 2010 and June 2011. Baseline characteristics were balanced between treatment technique groups (Table 1) with median age of 71 (IQR 66–74), median pre-hormone PSA of 9.5 ng/ml (IQR 6.8–12.40) and 12%, 77% and 11% low, intermediate and high risk respectively. At the time of the data snapshot for toxicity, median follow-up was 56.9 months (IQR 54.3–60.9) and for efficacy 73.3 (IQR 64.9–74.6) months.

**Table 1:** Baseline characteristics by treatment group (n = 293).

	No IGRT *N* = 48 *n* (%)	IGRT – S *N* = 137 *n* (%)	IGRT – R *N* = 108 *n* (%)
Randomisation option			
No IGRT v IGRT-S v IGRT-R	16 (33)	13 (9)	13 (12)
No IGRT v IGRT-S	32 (67)	32 (23)	–
IGRT-S v IGRT-R	–	92 (67)	95 (88)

Age (years)			
Median (IQR)	71 (66–73)	72 (66–75)	71 (67–75)
Range	57–80	53–82	54–80

Time from histological confirmation of prostate cancer to randomisation (wks)			
Median (IQR)	16 (13–25)	17 (13–27)	18 (13–25)
Range	6–350	3–278	4–265

T stage (clinical assessment)			
T1	16 (33)	46 (34)	43 (40)
T2	27 (56)	83 (61)	56 (52)
T3	5 (10)	8 (6)	9 (8)

Grading group(Gleason score)			
1 (3 + 3)	13 (27)	42 (31)	31 (29)
2 (3 + 4)	30 (63)	61 (45)	55 (51)
3 (4 + 3)	4 (8)	32 (23)	19 (18)
4 (4 + 4, 3 + 5, 5 + 3)	1 (2)	2 (1)	3 (3)

PSA (pre-hormone treatment) (ng/ml)			
Median (IQR)	9.5 (6.8–15.2)	9.7 (6.9–12.5)	8.3 (6.9–11.4)
Mean (SD)	11.1 (4.9)	10.3 (4.6)	9.1 (3.8)

PSA (ng/ml)			
0.0–4.99	2 (4)	13 (10)	12 (11)
5.0–9.99	23 (48)	57 (42)	60 (56)
10.0–19.99	20 (42)	65 (48)	36 (33)
20.0–49.99	3 (6)	2 (2)	0

NCCN Risk group			
Low	2 (4)	16 (12)	17 (16)
Medium	38 (79)	109 (80)	80 (74)
High	8 (17)	12 (9)	11 (10)

CHHiP treatment allocation			
74 Gy/37Fr	18 (38)	44 (32)	33 (31)
60 Gy/20Fr	15 (31)	48 (35)	37 (34)
57 Gy/19Fr	15 (31)	45 (33)	38 (35)

**Fig. 1:**
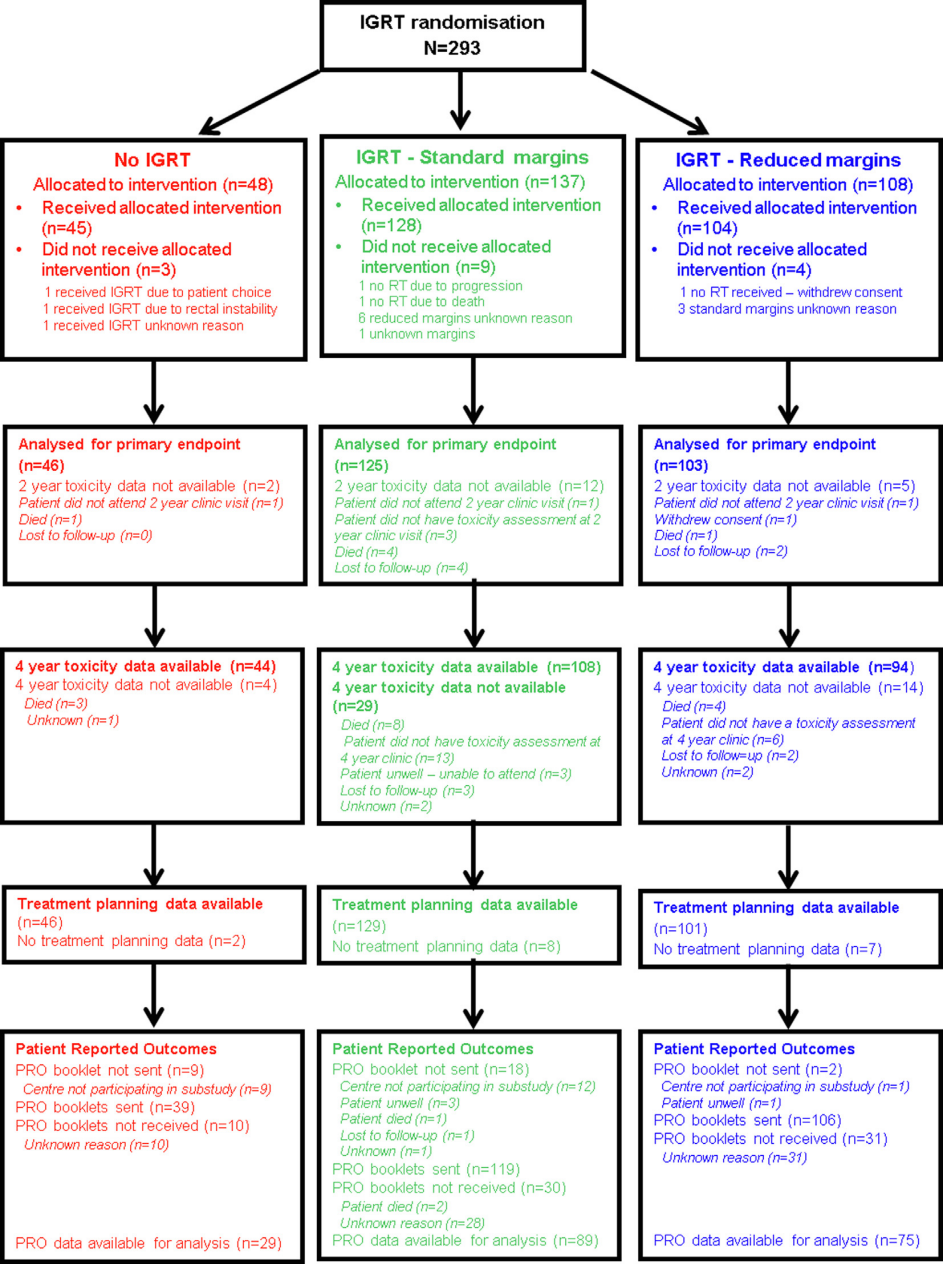
CONSORT diagram.

Three patients received no radiotherapy (one withdrew consent, one died and one biochemically progressed prior to radiotherapy). Adherence to randomly allocated treatment technique was high (Fig. 1): three no-IGRT patients received IGRT, nine IGRT-S patients did not receive standard CHHiP planning margins and four IGRT-R patients did not have reduced margins. Two-hundred and twenty-five patients had image guidance using fiducials, 11 patients were treated using TomoTherapy**®** and six patients using CT on rails.

**Fig. 2:**
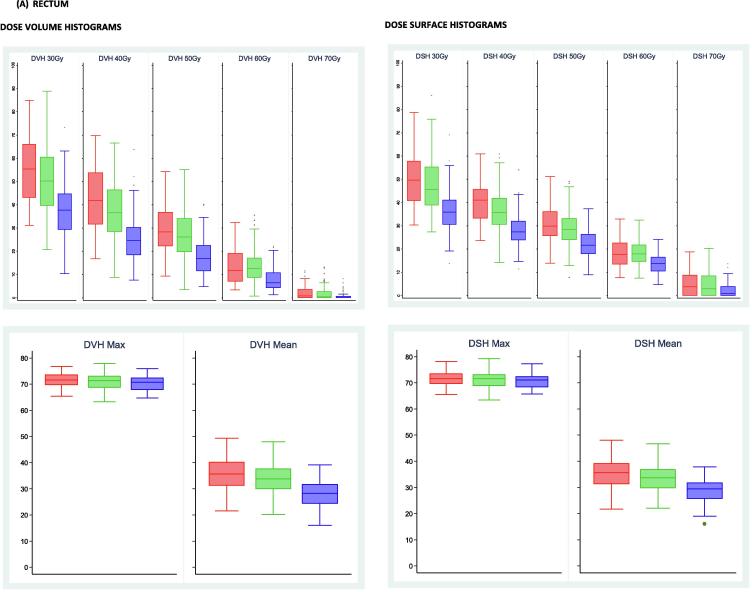

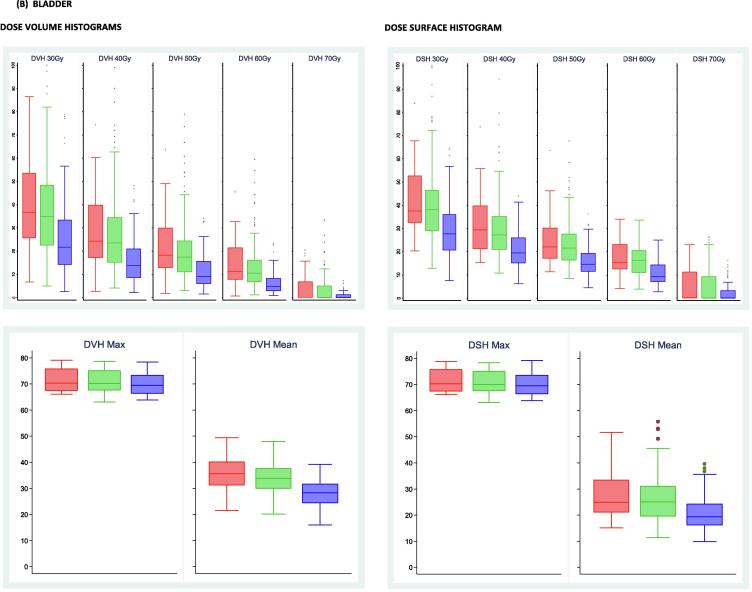
Boxplots illustrating dose volume and dose surface histograms, the calculated volume and surface percentages for rectum (A) and bladder (B) normal tissues by IGRT group and dose KEY: All doses are equivalent dose in 2 Gy fractions. Red boxplots represent patients treated with no IGRT, green boxplots are those patients treated with IGRT standard margins and blue boxplots are those patients treated with IGRT reduced margins.

Median (IQR) rectum volumes were 65 (59–77), 68 (56–86) and 67 (58–85) cm^3^ for the no-IGRT, IGRT-S and IGRT-R groups respectively. Corresponding figures for bladder volumes were 277 (200–379), 249 (167–375) and 281 (180–386) cm^3^ (Table S4). A summary of DVH and DSH for rectum and bladder by treatment group are shown in Fig. 2. Both rectal and bladder dose volume and surface percentages were consistently statistically lower in the IGRT-R compared to IGRT-S group (Table S5). Adherence to rectal dose constraints of 68% to 100% of prescribed dose and the bowel constraint was seen for 98% of patients, with all patients within the IGRT-R group achieving all these constraints (Table S6-S7).

**Fig. 3:**
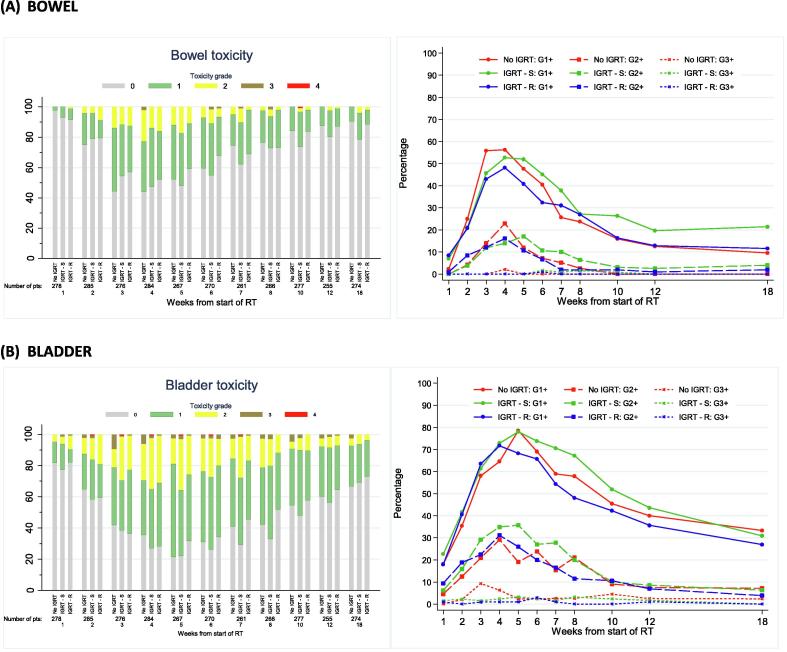
Acute RTOG bowel (A) and bladder (B) toxicity by timepoint and IGRT group. Distribution of grade and prevalence.

Toxicity associated with fiducial marker insertion was minimal with 19/190 (10%) reporting grade 1 and one patient reporting grade 2 haemorrhage. Six (3%) patients had an infection, three grade 1, two grade 2 and one grade 3. Worst RTOG bowel toxicity reported during 18 weeks from starting radiotherapy was grade ≥2 in 13/48 (27%), 38/135 (28%) and 26/107 (24%) of no-IGRT, IGRT-S and IGRT-R patients respectively. Corresponding figures for RTOG bladder grade ≥2 were 21/48 (44%), 71/135 (53%) and 48/107 (45%). By week 18, majority of toxicity had resolved with grade ≥2 bowel toxicity reported in 0% no-IGRT, 5% IGRT-S and 2% IGRT-R patients and RTOG bladder grade ≥2 reported in 3% no-IGRT, 8% IGRT-S and 4% IGRT-R patients (Fig. 3).

**Fig. 4:**
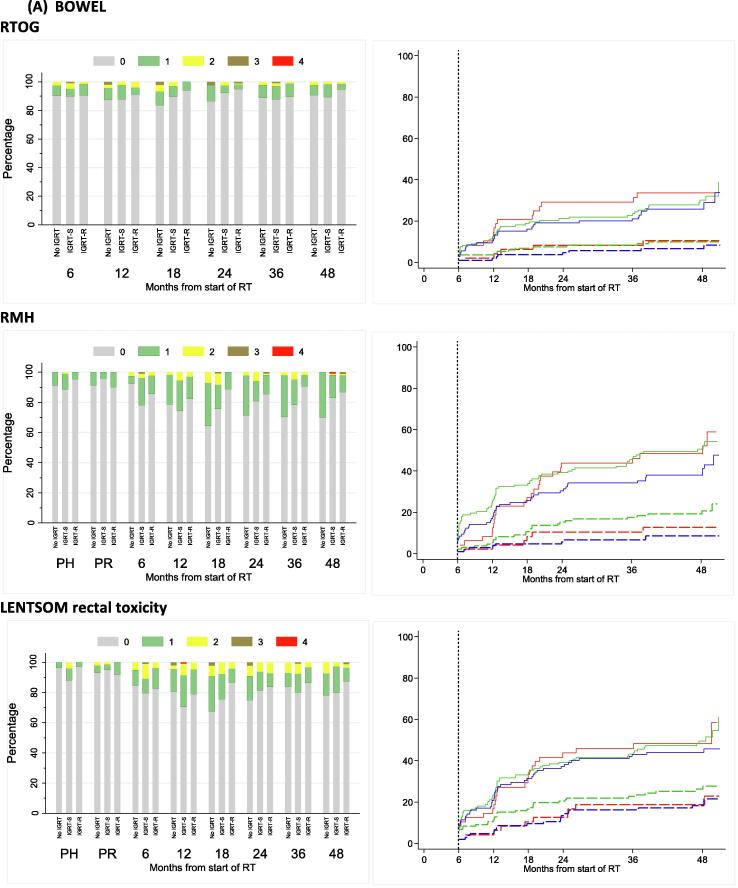

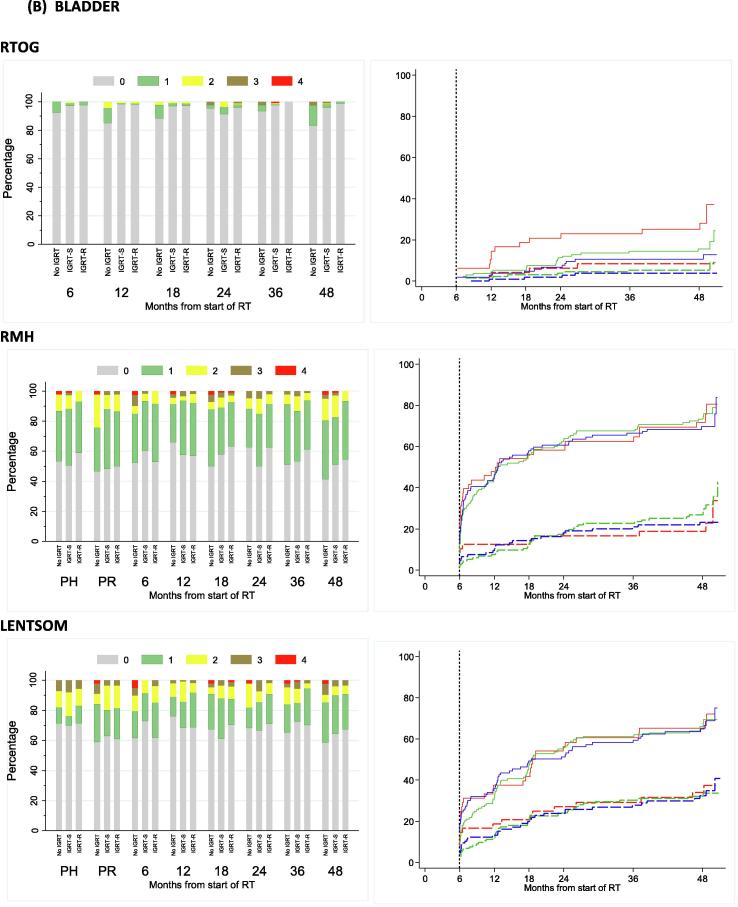
Bowel (A) and bladder (B) toxicity assessed using RTOG, RMH and LENTSOM by timepoint and IGRT group. Distribution and cumulative proportion with grade ≥1 and grade ≥2 radiation induced late toxicity. KEY: For cumulative proportion plots: red = No IGRT, Green = IGRT-S, Blue = IGRT-R. Solid lines indicated grade ≥1 events and dashed lines indicate grade ≥2 events NB. Late toxicity data have been included in stacked bar charts if they were reported within 6 weeks of the 6 month visit, within 3 months of the 12–24 month visits and within 6 months of the 36 and 48 month visits.

At two years, RTOG bowel and bladder toxicity was low across all treatment technique groups (Table S8) with 13 out of 274 (4.7%) patients assessed reporting any RTOG grade ≥2 toxicity, which was the primary endpoint of the substudy. The upper limits of 95% confidence intervals ruled out greater than 20% toxicity within each treatment technique group. Moderate to severe RTOG bowel toxicity was similar across treatment groups, with 1/46 (2%), 3/125 (2%) and 2/103 (2%) no-IGRT, IGRT-S and IGRT-R patients reporting grade ≥2 at 2 years. Cumulative proportion with grade ≥2 RTOG bowel toxicity reported to 2 years was 8.3% (95%CI 3.2–20.7), 8.3% (4.7–14.6%) and 5.8% (2.6–12.4%) for no-IGRT, IGRT-S and IGRT-R groups respectively (Fig. 4A and Table S9). RMH and LENTSOM scales showed similar low levels of moderate to severe bowel/rectum toxicity. RMH bowel grade ≥2 showed reduced toxicity in the IGRT-R group compared to IGRT-S with borderline statistical significance (HR = 0.39, 95%CI 0.18–0.83, p = 0.012).

Moderate to severe RTOG bladder toxicity was similar across treatment technique groups, with 1/46 (2%), 4/125 (3%) and 2/103 (2%) no-IGRT, IGRT-S and IGRT-R patients reporting grade ≥2 at 2 years. Cumulative proportion of RTOG bladder grade ≥2 toxicity by 2 years was low for all groups with the least toxicity reported in the IGRT-R group: 8.4 (3.2–20.8)%, 4.6 (2.1–9.9)% and 3.9 (1.5–9.9)% for no-IGRT, IGRT-S and IGRT-R groups respectively (Fig. 4B and Table S9). The RMH and LENTSOM scales reported higher incidences of bladder toxicity compared to RTOG but with a similar trend across groups. There was no evidence of significant differences between treatment technique groups.

A total of 193/265 (72.8%) PRO booklets were completed at a median of 50.3 months (IQR 47.8–52.0) from randomisation. Baseline characteristics were balanced between treatment technique groups and there were no significant differences between patients who did and did not complete the PRO booklet (Table S10). There was no evidence of any differences between treatment technique groups for EPIC or Vaizey summary scores (Table 2), with no suggestion of a worsening of Vaizey score with increased dose volume or dose surface at any dose level (Table S11). Median DVH and DSH values were calculated for patients whose EPIC bowel and urinary scores were below and above the threshold level previously defined (Table S3). There was a trend that patients whose score were below the cut-point had higher dose volume or surface levels (Fig. S1).

**Table 2:** Bowel and urinary domain scores using EPIC questionnaire and scoring system by treatment group at a median follow-up of 50.3 months (IQR: 47.8–52.0 months).

	No IGRT		IGRT-S		IGRT-R	
	No. of pts with data	Median (IQR)	No. of pts With data	Median (IQR)	No. of pts with data	Median (IQR)
Bowel function	29	96.4 (89.3–100)	89	92.9 (85.7–96.4)	75	96.4 (85.7–100)
*P*-value				0.210^1^		0.500^2^
Bowel bother	29	96.4 (87.5–100)	83	92.9 (85.7–100)	74	92.9 (85.7–100)
*P*-value				0.475^1^		0.909^2^
Bowel summary	29	94.6 (89.3–98.2)	84	94.6 (87.5–96.4)	74	92.9 (87.5–98.2)
*P*-value				0.218^1^		0.586^2^
Urinary function	28	97.5 (92.2–100)	87	100 (89.2–100)	75	95 (88.4–100)
*P*-value				0.941^1^		0.409^2^
Urinary bother	28	82.1 (74.1–93.8)	80	85.7 (71.4–92.9)	70	85.7 (75.9–92.9)
*P*-value				0.947^1^		0.456^2^
Urinary incontinence	27	100 (82.4–100)	79	93.8 (85.5–100)	71	93.8 (79.3–100)
*P*-value				0.985^1^		0.473^2^
Irritative/Obstructive	28	88.4 (78.6–96.0)	79	89.3 (80.7–92.9)	70	89.3 (85.7–96.4)
*P*-value				0.861^1^		0.454^2^
Urinary summary	27	88.9 (80.6–96.9)	79	88.9 (78.7–95.8)	71	89.6 (84–94.8)
*P*-value				0.945^1^		0.733^2^
Vaizey total score	27	4 (1–5.5)	84	4 (1–7)	72	4 (1–6)
				0.048^1^		0.821^2^

1 Comparison of No IGRT and IGRT-S groups.

2 Comparison of IGRT-S and IGRT-R groups.

Thirty-three patients had biochemical/clinical failure reported (4, 20 and 9 in no IGRT, IGRT-S and IGRT-R groups respectively) (Table S12 and Fig. S2). Five-year biochemical/clinical failure free survival was 91.1 (95% CI 77.9–96.6), 85.2 (95%CI 77.7–90.3) and 93.1 (95%CI 86.1–96.7) for no IGRT, IGRT-S and IGRT-R groups respectively. Fourteen patients had recommenced androgen deprivation therapy, nine had local recurrence, seven had lymph node/pelvic recurrence and seven had distant recurrence. Twenty-seven patients had died, three from prostate cancer, twenty-three from other reasons and one unknown.

## Discussion

We have demonstrated that implementation of IGRT was feasible in a multi-centre trial in the UK. Recruitment of patients was swift and completed within one year. Accrual peaked at 45 patient/month, with 16 radiotherapy centres participating. Subsequently IGRT has become part of the national guidelines recommended treatment pathway. This emphasises the importance of clinical trials as a vehicle to introduce advanced radiotherapy technology. Limitations of this substudy include its relatively small size, uneven randomisation between treatment technique groups and PRO assessment at a single time point. Yet, to our knowledge, this is the only randomised prospective study evaluating no-IGRT, IGRT and treatment margins using the same planning techniques.

We found minimal toxicity associated with insertion of fiducial markers. Dosimetric assessments showed that reduced margins in the IGRT-R group resulted in rectal and bladder volumes receiving 5–65 Gy being significantly lower (<0.0001) than using standard margins, this was also seen for surface dose. The mean dose to rectal surface in the IGRT-S group was 33.9(±5.1) Gy and similar to that previously reported of 34.4(±7.2) Gy using IG-IMRT [28] despite differences in dose prescription and margining techniques. As expected, mean dose to rectum in the IGRT-R group was significantly lower at 28.9(±4.2) Gy. Similarly, mean dose to bladder surface was 26.6(±9.2) Gy/20.5(±6.6)Gy for IGRT-S/IGRT-R groups respectively, both lower than previously reported (33.1 ± 10.9 Gy) [28]. Late GI toxicity was consistently reported less often using the three clinician based scores in the IGRT-R group. However, the improved rectal dosimetry did not translate into a statistically significant benefit in acute or late GI toxicity, with the possible exception of grade ≥2 RMH GI side-effects. This perhaps unexpected result may relate to the low level of side-effects seen in all randomised dose/fractionation groups in the main CHHiP trial which used strict normal tissue dose constraints and a SIB technique limiting dose to the seminal vesicles [10]. The lack of reduction in acute and late GU side effects may relate to similar doses to the urethra in all treatment technique groups. It may be the combination of dose/volume/fractionation employed in the trial has reached a plateau for radiotherapy side effects and other patient [29], radiogenomic [30] or microbiota [31] related factors become more important in determining residual symptoms. We believe it imprudent to extrapolate to treatments using higher doses, treating larger target volumes or using more extreme forms of hypofractionation where the clinical benefits of IGRT-R might be more apparent. Previous non-randomised studies comparing patient cohorts treated with IG-IMRT and 3D-CFRT [28], [29] have suggested improvements in grade ≥2 GU or GI side-effects using IGRT but are compromised by differences in planning and delivery techniques as well as differing dose constraints. Two randomised trials have evaluated daily versus weekly image guided radiotherapy. Tondel et al. reported no difference in acute patient reported outcomes, despite dosimetric advantages seen with daily CBCT and reduced margins, their late toxicity results are awaited [32]. A further study by de Crevoisier et al. showed no statistically significant difference in recurrence free survival, with also no difference in acute toxicity [33]. A statistically significant difference in late rectal toxicity and improvement in biochemical recurrence was reported, albeit with a short median follow up of 4.1 years. We found no evidence to suggest that IGRT was associated with a reduction in disease control. IGRT may increase accuracy and reduce the chance of underdosage in the target volume, alternatively however it is possible that the reduced margins might lower inadvertent dose outside the prostate which has been suggested as a cause of treatment failure [34].

The IGRT experience gained within this study has facilitated development of a new national trial PIVOTALboost (CRUK/16/018) in which all patients receive IG-IMRT and are randomised to receive pelvic lymph node IMRT or MR-directed dominant lesion boosts using hypofractionated schedules as in the CHHiP study.

We have shown it is feasible to introduce prostate IGRT in a national randomised trial and that reduced margins translate into dosimetric benefits. Overall side effect profiles were low with and without IGRT in the CHHiP trial and this substudy.

## Funding

Cancer Research UK, Department of Health, and the National Institute of Health Research Cancer Research Network.
