# Supplementary material for: A randomised assessment of image guided radiotherapy within a phase 3 trial of conventional or hypofractionated high dose intensity modulated radiotherapy for prostate cancer
Source: Radiother Oncol. 2020 Jan;142:62–71. doi: 10.1016/j.radonc.2019.10.017 (PMC7005673; doi:10.1016/j.radonc.2019.10.017)
Supplement: Supplementary data 1 [file mmc1.doc]

**CHHiP IGRT manuscript – Supplementary information**

Table S1: Margin definition and target isodose levels prescriptive to the planning

|  | **No IGRT and IGRT - S** | | **IGRT - R** | |
| --- | --- | --- | --- | --- |
| Target Isodoses | Anterior, right, left, superior, inferior | Posterior | Anterior, right, left, superior, inferior | Posterior |
| GTV 1 to PTV 1  (>80% median to PTV1 – PTV 2) | 10 mm | 10 mm | 6 mm | 6 mm |
| GTV 2 to PTV 2  (>96% median to PTV2 – PTV 3) | 10 mm | 5 mm | 6 mm | 3 mm |
| GTV 3 to PTV 3  (100% (+1%) median) | 5 mm | 0 mm | 3 mm | 0 mm |

GTV 1 = prostate and base of SV; GTV 2 = prostate +/- base of SV; GTV 3 = prostate

**Table S2:** Normal tissue dose constraints

|  | Prescribed Dose (Gy) | | | Dose (%) | Max Vol (% or cc) | |
| --- | --- | --- | --- | --- | --- | --- |
|  | 74Gy/37F | 60Gy/20F | 57Gy/19F |  | Optimal | Mandatory |
| Rectum | 30  40  50  60  65  70  74 | 24.3  32.4  40.5  48.6  52.7  56.8  60 | 23.1  30.8  38.5  46.2  50.1  53.9  57 | 41  54  68  81  88  95  100 | 80%  70% | 60%  50%  30%  15%  3% |
| Bladder | 50  60  74 | 40.5  48.6  60 | 38.5  46.2  57 | 68  81  100 |  | 50%  25%  5% |
| Femoral heads | 50 | 40.5 | 38.5 | 68 |  | 50% |
| Bowel | 50 | 40.5 | 38.5 | 68 |  | 17cc |
| Urethral bulb | 50  60 | 40.5  48.6 | 38.5  46.2 | 68  81 | 50%  10% |  |

**Table S3: EPIC bowel domain mean and standard deviation scores from the CHHiP QoL substudy and the calculated threshold dose**

| **EPIC** | **Summary** | | **Function** | | **Bother** | |
| --- | --- | --- | --- | --- | --- | --- |
|  | N | Mean(SD) | N | Mean(SD) | N | Mean(SD) |
| **Bowel** | 338 | 93.6 (7.79) | 334 | 93.3 (8.20) | 340 | 94.1 (9.29) |
| **Threshold score** |  | 89.7 |  | 89.2 |  | 89.5 |

**Table S4: Treatment** planning data

|  | **No IGRT** | **IGRT - S** | **IGRT - R** |
| --- | --- | --- | --- |
| **Rectal volume**  **Median (IQR)** | 65 (59-77) | 68 (56-86) | 67 (58-85) |
| **Rectal surface**  **Median (IQR)** | 138 (122-152) | 136 (122-159) | 135 (115-154) |
| **Bladder volume**  **Median (IQR)** | 277 (200-379) | 249 (167-375) | 281 (180-386) |
| **Bladder surface**  **Median (IQR)** | 263 (210-343) | 274 (220-381) | 310 (227-435) |
| **PTV1**  **Median (IQR)** | 220 (190-252) | 194 (165-224) | 121 (98-144) |
| **PTV2**  **Median (IQR)** | 146 (130-171) | 139 (120-173) | 89 (70-109) |
| **PTV3**  **Median (IQR)** | 86 (73-102) | 84 (69-106) | 61 (47-77) |

**Table S5: P-values for statistical comparison of dose volume and dose surface histograms for No IGRT vs IGRT-S and IGRT-S vs IGRT-R. All doses are in equivalent dose in 2Gy fractions.**

|  | **No IGRT vs IGRT-S** | **IGRT-S vs IGRT-R** |
| --- | --- | --- |
| **Rectal DVH**  **5Gy**  **10Gy**  **15Gy**  **20Gy**  **25Gy**  **30Gy**  **35Gy**  **40Gy**  **45Gy**  **50Gy**  **55Gy**  **60Gy**  **65Gy**  **70Gy**  **Min**  **Max**  **Mean** | <0.0001  0.035  0.032  0.281  0.412  0.101  0.027  0.022  0.049  0.281  0.949  0.732  0.999  0.450  0.008  0.383  0.097 | <0.0001  <0.0001  <0.0001  <0.0001  <0.0001  <0.0001  <0.0001  <0.0001  <0.0001  <0.0001  <0.0001  <0.0001  <0.0001  0.003  <0.0001  0.038  <0.0001 |
| **Rectal DSH**  **5Gy**  **10Gy**  **15Gy**  **20Gy**  **25Gy**  **30Gy**  **35Gy**  **40Gy**  **45Gy**  **50Gy**  **55Gy**  **60Gy**  **65Gy**  **70Gy**  **Min**  **Max**  **Mean** | 0.082  0.057  0.110  0.884  0.686  0.185  0.046  0.023  0.019  0.158  0.873  0.517  0.598  0.501  0.007  0.551  0.174 | <0.0001  <0.0001  <0.0001  <0.0001  <0.0001  <0.0001  <0.0001  <0.0001  <0.0001  <0.0001  <0.0001  <0.0001  <0.0001  0.003  <0.0001  0.056  <0.0001 |
| **Bladder DVH**  **5Gy**  **10Gy**  **15Gy**  **20Gy**  **25Gy**  **30Gy**  **35Gy**  **40Gy**  **45Gy**  **50Gy**  **55Gy**  **60Gy**  **65Gy**  **70Gy**  **Min**  **Max**  **Mean** | 0.876  0.640  0.491  0.350  0.280  0.331  0.308  0.324  0.306  0.351  0.364  0.422  0.477  0.279  0.305  0.337  0.334 | <0.0001  <0.0001  <0.0001  <0.0001  <0.0001  <0.0001  <0.0001  <0.0001  <0.0001  <0.0001  <0.0001  <0.0001  <0.0001  0.006  0.0003  0.027  <0.0001 |
| **Bladder DSH**  **5Gy**  **10Gy**  **15Gy**  **20Gy**  **25Gy**  **30Gy**  **35Gy**  **40Gy**  **45Gy**  **50Gy**  **55Gy**  **60Gy**  **65Gy**  **70Gy**  **Min**  **Max**  **Mean** | <0.0001  0.996  0.727  0.434  0.290  0.206  0.238  0.283  0.346  0.416  0.553  0.667  0.588  0.359  0.206  0.330  0.475 | <0.0001  <0.0001  <0.0001  <0.0001  <0.0001  <0.0001  <0.0001  <0.0001  <0.0001  <0.0001  <0.0001  <0.0001  <0.0001  0.043  0.0001  0.067  <0.0001 |

Table S6: Descriptive statistics given by IGRT treatment allocation for rectum and bowel constraints

|  |  |  | N  Median (IQR)  Range | N  Median (IQR)  Range | N  Median (IQR)  Range |
| --- | --- | --- | --- | --- | --- |
|  | Dose (%) of  prescribed Dose | Max Vol  (% or cc) | **No IGRT** | **IGRT-S** | **IGRT-R** |
| **Rectum** | 68  81  88  95  100 | 60%  50%  30%  15%  3% | 48  41.9 (32.0-51.2)  15.6-62.8  48  25.2 (18.1-32.5)  7.9-53.7  48  13.8 (9.8-18.1)  3.6-37.4  48  5.4 (3.2-8.9)  0.6-24.6  47  0 (0-0)  0-3.4 | 135  36.4 (27.5-47.2)  8.6-63.6  133  23.3 (16.5-31.9)  3.1-47.6  133  14.5 (10.1-20.6)  1.1-30.4  133  5.6 (2.8-7.7)  0.7-15.0  133  0 (0-0)  0-2.1 | 107  23.9 (16.2-32.3)  2.5-59.7  104  15.1 (8.3-20.5)  4.2-44.7  104  8.0 (4.4-11.9)  2.2-25.9  104  2.1 (1.0-4.0)  0.1-12.4  101  0 (0-0)  0-1.5 |
| **Bowel** | 68 | 17cc | 24  0.6 (0-0)  0-95.5 | 98  0.4 (0-0)  0-33.3 | 81  0 (0-0)  0-15.8 |

A patient has been defined as adhering to rectum dose constraints if their maximum percentage volume was less than or equal to that specified in the table above for 68-100% of prescribed dose.

Table S7: Descriptive statistics given by IGRT treatment allocation for bladder constraints

|  |  |  | N  Median (IQR)  Range | N  Median (IQR)  Range | N  Median (IQR)  Range |
| --- | --- | --- | --- | --- | --- |
|  | Dose (%) of  prescribed Dose | Max Vol  (%) | **No IGRT** | **IGRT-S** | **IGRT-R** |
| **Bladder** | 68  81  100 | 50%  25%  5% | 48  23.6 (16.6-37.3)  10.6-75.8  48  16.6 (12.0-30.6)  5.9-62.8  48  2.4 (0.8-4.0)  0.1-10.2 | 135  21.4 (15.2-31.5)  5.2-83.4  135  16.0 (10.7-23.4)  0.3-71.7  134  1.7 (0.6-3.0  0-16.7 | 107  11.9 (8.5-19.4)  3.6-43.4  105  7.9 (5.5-13.0)  0.3-33.3  104  0.2 (0-0.5)  0-5.8 |

A patient has been defined as adhering to bladder dose constraints if their maximum percentage volume was less than or equal to that specified in the table above for 68-100% of prescribed dose.

A total of 43 (out of 286 with data) patients missed at least one of the 3 bladder dose constraints:

19 patients missed one constraint; 16 patients missed two bladder constraints; 8 patients missed all three bladder constraints

Table S8: Number and proportion with RTOG grade>=2 toxicity at 2 years with 95% confidence intervals - by treatment group and randomisation strategy

| **Randomisation strategy** | **No IGRT v IGRT - S v IGRT - R** | **No IGRT v IGRT - S** | **IGRT - S v IGRT - R** | **Overall** |
| --- | --- | --- | --- | --- |
| **RTOG BLADDER OR BOWEL** | | | | |
| **No IGRT**  n/ N  Proportion (95%CI) | 0 / 15  0 (0-21.8)% | 2 / 30  6.7 (0.8-22.1)% | - | 2 / 46  4.3 (0.5-14.8)% |
| **IGRT - S**  n / N  Proportion (95%CI) | 0 / 10  0 (0-30.8)% | 3 / 29  10.3 (2.2-27.4)% | 4 / 86  4.7 (1.3-11.5%) | 7 / 125  5.6 (2.3-11.2)% |
| **IGRT - R**  n / N  Proportion (95%CI) | 3 / 12  25 (5.5-57.2)% | - | 1 / 91  1.1 (0.1-6.0)% | 4 / 103  3.9 (1.1-9.6)% |
| **RTOG BOWEL** | | | | |
| **No IGRT**  n / N  Proportion (95%CI) | 0 / 15  0 (0-21.8)% | 1 / 30  3.3 (0.1-17.2)% | - | 1 / 46  2.2 (0.1-11.5)% |
| **IGRT - S**  n / N  Proportion (95%CI) | 0 / 10  0 (0-30.8)% | 1 / 29  3.4 (0.1-17.8)% | 2 / 86  2.3 (0.3-8.1)% | 3 / 125  2.4 (0.5-6.9)% |
| **IGRT - R**  n / N  Proportion (95%CI) | 2 / 12  16.7 (2.1-48.4)% | - | 0 / 91  0 (0-4.0)% | 2 / 103  1.9 (0.2-6.8)% |
| **RTOG BLADDER** | | | | |
| **No IGRT**  n / N  Proportion (95%CI) | 0 / 15  0 (0-21.8) | 1 / 30  3.3 (0.1-17.2) | - | 1 / 46  2.2 (0.1-11.5)% |
| **IGRT - S**  n / N  Proportion (95%CI) | 0 / 10  0 (0-30.8)% | 2 / 29  6.8 (0.8-22.8)% | 2 / 86  2.3 (0.3-8.1)% | 4 / 125  3.2 (0.9-8.0)% |
| **IGRT - R**  n / N  Proportion (95%CI) | 1 / 12  8.3 (0.2-38.5) | - | 1 / 91  1.1 (0.1-6.0)% | 2 / 103  1.9 (0.2-6.8)% |

NB. There was one patient who was originally from an IGRT-S v IGRT-R centre but was randomised to No IGRT group following the staggered closure of recruitment so the IGRT-S v IGRT-R centre changed to a No IGRT v IGRT-S v IGRT-R centre and randomised one patient to No IGRT group . This patient has been included in the overall No IGRT group figures but not in the by randomisation strategy figures.

Table S9: Hazard ratios and cumulative proportion with G1+, G2+ and G3+ bowel and bladder toxicity at 2 years assessed by RTOG, RMH and LENTSOM scoring systems

|  | **No IGRT v IGRT-S** | | | | **IGRT-S v IGRT-R** | | | | **Cumulative proportion with events at 2 years^3^ (95%)** | | | | |
| --- | --- | --- | --- | --- | --- | --- | --- | --- | --- | --- | --- | --- | --- |
|  | **HR (95% CI)** | | | **P^1^** | **HR (95% CI)** | | **P^1^** | | **No IGRT** | | **IGRT-S** | | **IGRT=RC** |
| **BOWEL** | |  |  | | |  | |  | |  | |  | |
| **RTOG** | |  |  | | |  | |  | |  | |  | |
| Grade>=1  64 events^2^ | 0.85 (0.48-1.53) | | | 0.596 | 0.85 (0.53-1.35) | | 0.483 | | 29.2 (18.4-44.2) | | 22.0 (15.8-30.0) | | 20.0 (13.6-29.1) |
| Grade>=2  21 events | 0.95 (0.34-2.67) | | | 0.928 | 0.68 (0.28-1.62) | | 0.381 | | 8.3 (3.2-20.7) | | 8.3 (4.7-14.6) | | 5.8 (2.6-12.4) |
| Grade>=3  4 events | 0.36 (0.05-2.56) | | | 0.287 | 0.61 (0.06-6.71) | | 0.682 | | 4.2 (1.1-15.7) | | 0.8 (0.1-5.2) | | 1.0 (0.1-6.6) |
| **RMH** | | | | | | | | | | | | | |
| Grade>=1  112 events^2^ | 1.06 (0.67-1.69) | | | 0.786 | 0.71 (0.48-1.03) | | 0.071 | | 43.8 (31.1-58.8) | | 41.5 (33.7-50.4) | | 34.2 (26.0-44.1) |
| Grade>=2  34 events | 1.69 (0.70-4.10) | | | 0.239 | 0.39 (0.18-0.83) | | 0.012 | | 10.4 (4.5-23.2) | | 16.7 (11.4-24.3) | | 6.7 (3.2-13.5) |
| Grade>=3  3 events | - | | | 0.325 | 0.79 (0.13-4.76) | | 0.799 | | - | | 1.5 (0.04-5.9) | | 1.0 (0.01-6.6) |
| **LENTSOM** | | | | | | | | | | | | | |
| Grade>=1  120 events^2^ | 0.99 (0.63-1.60) | | | 0.999 | 0.83 (0.57-1.21) | | 0.331 | | 45.9 (33.1-60.9) | | 41.5 (33.7-50.4) | | 41.1 (32.4-51.2) |
| Grade>=2  55 events | 1.36 (0.68-2.75) | | | 0.385 | 0.69 (0.40-1.19) | | 0.181 | | 18.8 (10.3-33.0) | | 21.9 (15.8-30.0) | | 16.3 (10.4-24.9) |
| Grade>=3  5 events | 0.54 (0.09-3.26) | | | 0.498 | 0.81 (0.14-4.85) | | 0.818 | | 4.2 (1.1-15.7) | | 1.5 (0.4-5.9) | | 0.9 (0.1-6.5) |
| **BLADDER** | |  |  | | |  | |  | |  | |  | |
| **RTOG** | |  |  | | |  | |  | |  | |  | |
| Grade>=1  39 events | 0.51 (0.26-0.99) | | | 0.044 | 0.67 (0.33-1.35) | | 0.255 | | 22.9 (13.4-37.6) | | 12.9 (8.2-19.9) | | 10.6 (6.0-18.3) |
| Grade>=2  14 events | 0.69 (0.21-2.29) | | | 0.538 | 0.62 (0.19-2.07) | | 0.436 | | 8.4 (3.2-20.8) | | 4.6 (2.1-9.9) | | 3.9 (1.5-9.9) |
| Grade>=3  2 events | 0.66 (0.06-7.34) | | | 0.735 | 0.61 (0.06-6.75) | | 0.686 | | 2.1 (0.3-14.2) | | 0 | | 1.0 (0.1-6.6) |
| **RMH** | | | | | | | | | | | | | |
| Grade>=1  187 events | 0.95 (0.65-1.38) | | | 0.774 | 0.97 (0.72-1.31) | | 0.828 | | 62.5 (49.1-75.9) | | 67.7 (59.7-75.4) | | 63.6 (54.5-72.7) |
| Grade>=2  57 events | 1.34 (0.69-2.62) | | | 0.385 | 0.74 (0.44-1.22) | | 0.231 | | 16.7 (8.7-30.6) | | 22.0 (15.8-30.0) | | 19.1 (12.8-28.0) |
| Grade>=3  21 events | 0.90 (0.32-2.53) | | | 0.840 | 0.66 (0.26-1.66) | | 0.375 | | 10.4 (4.5-23.2) | | 7.6 (4.2-13.7) | | 5.8 (2.6-12.4) |
| **LENTSOM** | | | | | | | | | | | | | |
| Grade>=1  169 events | 0.95 (0.64-1.42) | | | 0.815 | 1.04 (0.76-1.42) | | 0.811 | | 60.5 (47.1-74.2) | | 60.4 (52.2-68.8) | | 56.3 (47.0-65.9) |
| Grade>=2  81 events | 0.89 (0.51-1.56) | | | 0.678 | 1.01 (0.65-1.58) | | 0.958 | | 29.2 (18.5-44.3) | | 28.0 (21.1-36.5) | | 25.8 (18.5-35.4) |
| Grade>=3  27 events | 1.16 (0.42-3.15) | | | 0.776 | 0.97 (0.47-2.00) | | 0.932 | | 8.3 (3.2-20.7) | | 9.2 (5.3-15.6) | | 8.6 (4.6-15.9) |

^1^ Assessed using the non-stratified log-rank test; ^2^ Total number of events reported is up to 2 years (27 months); ^3^ Estimated at 27 months

Table S10: Baseline characteristics for PRO responders and non-responders

|  | PRO responder  N=193 | PRO non-responder  N=71 | P-value |
| --- | --- | --- | --- |
| Age at registration (years) |  |  |  |
| Median (IQR) | 71 (67-74) | 72 (65-75) | 0.83 |
| Range | 53-80 | 55-82 |  |
| Time from histological confirmation of prostate cancer to randomisation (wks)  Median (IQR)  Range | 17 (13-25)  4-278 | 19 (13-28)  6-126 | 0.98 |
| T stage (clinical assessment) |  |  |  |
| T1a/T1b/T1c/T1x | 76 (39) | 21 (30) | 0.10 |
| T2a/T2b/T2c/T2x | 108 (56) | 37 (52) |  |
| T3a/T3x | 9 (5) | 13 (18) |  |
| Grading (Gleason score) |  |  |  |
| 1 (3+3) | 53 (27) | 21 (30) | 0.45 |
| 2 (3+4)  3 (4+3) | 98 (51)  38 (20) | 37 (52)  13 (18) |  |
| 4 (4+4, 3+5, 5+3) | 4 (2) | 0 |  |
| PSA (pre-hormone treatment) (ng/ml) |  |  |  |
| Median (IQR) | 9.2 (6.6-12.5) | 9.1 (7.1-11.6) |  |
| Mean (SD) | 10.1 (4.5) | 0.5-18.6 |  |
| PSA (ng/ml) |  |  |  |
| 0.0<5 | 17 (9) | 8 (11) | 0.35 |
| 5.0<10 | 92 (48) | 35 (49) |  |
| 10.0<20 | 80 (41) | 28 (39) |  |
| 20.0<50 | 4 (2) | 0 |  |
| NCCN Risk group |  |  |  |
| Low | 24 (12) | 9 (13) | 0.67 |
| Medium | 153 (79) | 54 (76) |  |
| High | 16 (8) | 8 (11) |  |
| CHHiP treatment allocation  No IGRT  IGRT-S  IGRT-R | 29 (15)  89 (46)  75 (39) | 10 (13)  30 (42)  31 (44) | 0.55 |
| Co-morbidities  Diabetes  Hypertension  Inflammatory bowel disease or diverticular disease  Previous pelvic surgery  Symptomatic haemorrhoids (12 months prior to randomisation)  Previous TURP | 20 (11)  73 (41)  10 (6)  16 (9)  12 (7)  19 (11 | 8 (11)  25 (39)  2 (3)  4 (7)  3 (5)  3 (5) | 0.82  0.88  0.74  0.79  0.76  0.21 |

**Table S11: Percentage volume and surface receiving each dose level according to Vaizey scores categorised as tertiles (Ter1=Vaizey score 0-4; Ter2=Vaizey total 5-7; Ter3=Vaizey score 7-19). All doses are equivalent dose in 2Gy fractions.**

|  | **0 Gy** | **5 Gy** | **10 Gy** | **15 Gy** | **20 Gy** | **25 Gy** | **30 Gy** | **35 Gy** | **40 Gy** | **45 Gy** | **50 Gy** | **55 Gy** | **60 Gy** | **65 Gy** | **70 Gy** | **Volume** | **Min** | **Max** | **Mean** |
| --- | --- | --- | --- | --- | --- | --- | --- | --- | --- | --- | --- | --- | --- | --- | --- | --- | --- | --- | --- |
| **Dose Volume Histogram** | | | | | | | | | | | | | | | | | | | |
| **Ter1** | 100 | 89.1 | 86.1 | 81.8 | 72.2 | 57.3 | 42.6 | 34.7 | 30.1 | 26.1 | 22.5 | 17.3 | 10.9 | 5.4 | 0.3 | 65.8 | 1.3 | 71.5 | 31.7 |
| **Ter2** | 100 | 90.3 | 85.8 | 81.3 | 69.9 | 57.1 | 46.5 | 39.2 | 32.9 | 28.6 | 23.4 | 17.1 | 9.3 | 3.7 | 0.1 | 65.6 | 1.1 | 70.6 | 31.7 |
| **Ter3** | 100 | 89.5 | 85.0 | 81.7 | 73.7 | 57.6 | 43.2 | 36.2 | 30.6 | 26.3 | 22.0 | 17.7 | 11.1 | 5.5 | 0.3 | 69.2 | 1.2 | 71.1 | 31.8 |
| **Dose Surface Histogram** | | | | | | | | | | | | | | | | | | | |
| **Ter1** | 100 | 88.0 | 83.2 | 79.9 | 67.6 | 52.6 | 40.2 | 35.3 | 31.9 | 29.4 | 26.7 | 21.6 | 16.2 | 11.5 | 2.9 | 135.7 | 1.3 | 71.7 | 32. 4 |
| **Ter2** | 100 | 88.6 | 84.6 | 78.5 | 66.6 | 53.7 | 42.1 | 36.3 | 32.9 | 30.0 | 26.8 | 22.4 | 16.1 | 10.0 | 0.6 | 129.4 | 1.1 | 70.7 | 32.7 |
| **Ter3** | 100 | 87.8 | 82.7 | 77.6 | 67.0 | 51.0 | 41.1 | 35.7 | 31.5 | 28.6 | 25.0 | 21.2 | 16.7 | 11.3 | 2.1 | 138.5 | 1.2 | 71.3 | 31.6 |

**Figure S1: Boxplots showing along the top row the dose volume histograms for patients whose EPIC bowel (A) and urinary (B) domain scores were above or below the defined thresholds. The dose surface histograms are shown along the bottom row for those patients whose EPIC bowel (A) and urinary (B) domain scores were above or below the defined thresholds. All doses are equivalent dose in 2Gy fractions.**

**(A)**


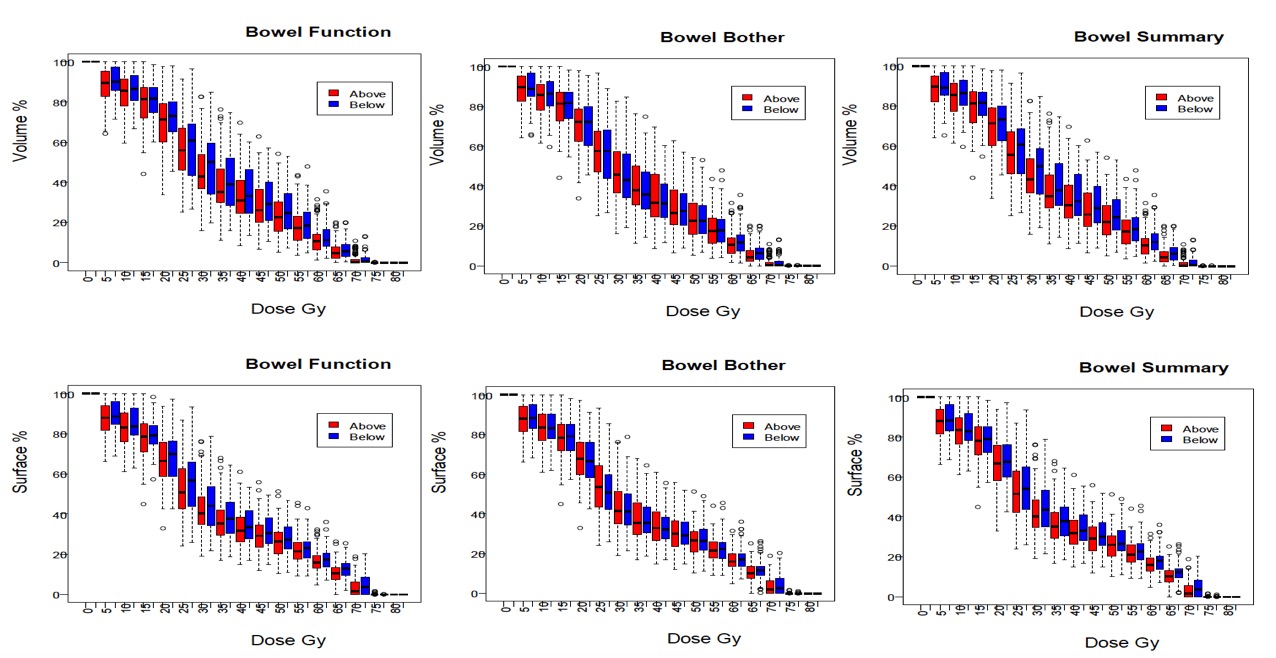


**(B)**


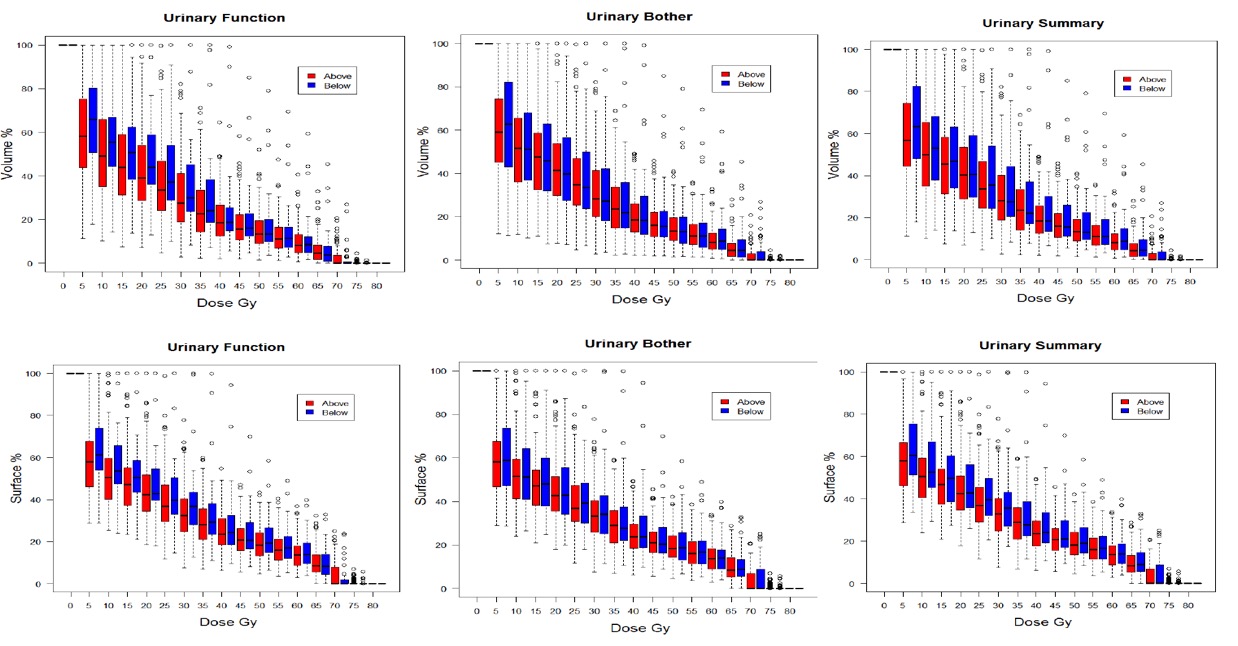


**Table S12: Efficacy data by IGRT substudy group**

|  | **No IGRT**  **N=48** | **IGRT – S**  **N=137** | **IGRT – R**  **N=108** |
| --- | --- | --- | --- |
| **Biochemical/clinical failure free survival**  N (events)  5yr KM estimate (95% CI) | 4 (8)  91.1 (77.9-96.6) | 20 (15)  85.2 (77.7-90.3) | 9 (8)  93.1 (86.1-96.7) |
| **Recommenced androgen deprivation**  n (%) | 0 | 11 (8) | 3 (3) |
| **Local recurrence**  n (%) | 0 | 7 (5) | 2 (2) |
| **Lymph node/pelvic recurrence**  n (%) | 1 (2) | 5 (4) | 1 (1) |
| **Distant recurrence**  n (%) | 1 (2) | 5 (4) | 1 (1) |
| **Died**  n (%)  **Cause of death:**  Prostate cancer related  Not related to prostate cancer  Unknown | 5 (10)  0  4  1 | 15 (11)  3  12  0 | 7 (7)  0  7  0 |

NB. Events are not mutually exclusive

**Figure S2 Kaplan Meier for biochemical/clinical failure free survival by IGRT substudy group**

**Radiotherapy Quality Assurance**

IGRT quality assurance (QA) programme was developed as an adjunct to the main radiotherapy trial QA to accredit centres to participate in the CHHiP IGRT substudy. The primary aim of the QA was to audit compliance with the IGRT requirements of the trial protocol.

The QA exercises comprised:

- Completion of a process document, to review and verify the centres’ IGRT techniques and processes
- Measurements to assess the accuracy of the imaging and corrections applied (accuracy of couch repositioning and kV/MV isocentre alignments).
- Collection of daily isocentre displacement data for all patients, to assess the feasibility of utilising data from multiple manufacturers’ equipment.

| Equipment | No. of centres | Imaging techniques |
| --- | --- | --- |
| Elekta XVI | 3 | kV cone beam 360°  kV orthogonal images |
| Varian OBI | 8 | kV cone beam 360°  kV orthogonal images |
| Elekta a-Si portal imager | 1 | MV orthogonal images |
| Varian a-Si portal imager | 5 | MV orthogonal images |
| Somatom CT on rails | 1 | kV CT scan |
| Tomotherapy | 1 | MV CT scan |
| ExacTrac | 1 | kV orthogonal images |

Table 1: Imaging procedures used by CHHiP IGRT centres

7 centres performed automatic couch repositioning, with a zero tolerance. The other 12 centres corrected for any set-up errors ≥2mm. 6 centres performed post-correction imaging, and 2 centres performed post-treatment imaging.

Issues identified during the QA process for 19 centres approved for the CHHiP IGRT substudy included:

- Whether to permit the use of a planning MR scan, fused with the planning CT images, for outlining the prostate CTV. Prostates outlined using MR imaging often have a smaller volume than those outlined using CT images. This was potentially an issue for the CHHiP IGRT study since the experimental arm used reduced PTV margins, and outlining the CTV using MR could have the effect of reducing the effective margin still further. A recommendation was therefore made that MR fusion for prostate outlining would be permitted for those few centres who had already introduced MR/CT fusion into their routine practice, but that the prostate apex should be very adequately outlined.
- Whether an action level for post-correction images should be applied. Post-correction images were mandatory for centres using manual couch repositioning equipment to ensure that the correction was applied in the right direction. No specific action level was set in the protocol, whereas an action level of 2mm was applied for the pre-treatment imaging.
- Imaging doses reported for different imaging techniques varied significantly in both value and dose descriptor, which made them difficult to interpret and compare.
- Set up displacements were not displayed or stored consistently between manufacturers. Some systems reported the physical couch shift required (e.g. -0.5cm vertical), while others converted this to a displacement relative to the patient position (e.g. 0.5cm anterior). Some systems did not store this information, hence these data were not available for retrospective analysis. Information was not recorded on the timing of the image (i.e. pre- or post-correction), or whether systematic shifts were applied.
- The level of IGRT experience had an impact of the number and grade of radiographers who were performing the on-line image registration in each centre.
